# ACCESS TO DENTAL CARE AND ORAL HEALTH INEQUITIES AMONG DIVERSE POPULATIONS IN THE UNITED STATES AND CHINA

**DOI:** 10.1093/geroni/igad104.0049

**Published:** 2023-12-21

**Authors:** Xiang Qi, Xi Chen, Elisa Ghezzi

## Abstract

Although the oral health status of the world population has improved over the years, structural and societal inequities in dental care and oral health are pervasive with older adults. This symposium discusses findings from three studies that examine structural and societal determinants of dental care and oral health inequities among diverse populations in the US and China. Mao’s study investigated correlates of dental care utilization among informal dementia caregivers in the US. Findings demonstrate that spousal caregivers and those who provide intensive care are less likely to visit a dentist. These findings suggest that dementia caregiver support intervention strategies should target these populations to improve dental care-seeking behaviors. Qi’s study examined the population attributable fraction of poor oral health on incident dementia in comparison with other modifiable risk factors. This study found that poor oral health, including edentulism and oral health problems, is a significant risk factor for incident dementia. The study highlights the need to integrate dental care as an integral component of a comprehensive approach to promoting cognitive health. Qu’s study explored social structural disparities in dental care utilization among adults in China’s megacities. This study revealed that social structural factors, such as hukou status, occupational status, and city of residence, are associated with disparities in dental care utilization. Overall, these studies shed light on important factors that impact dental care utilization and oral health. Addressing disparities in dental care utilization and oral health should be a priority in promoting overall health and well-being, particularly among vulnerable populations.

